# Consensus on the clinical management, screening‐to‐treat, and surveillance of *Helicobacter pylori* infection to improve gastric cancer control on a nationwide scale

**DOI:** 10.1111/hel.12368

**Published:** 2017-01-08

**Authors:** Bor‐Shyang Sheu, Ming‐Shiang Wu, Cheng‐Tang Chiu, Jing‐Chuan Lo, Deng‐Chyang Wu, Jyh‐Ming Liou, Chun‐Ying Wu, Hsiu‐Chi Cheng, Yi‐Chia Lee, Ping‐I Hsu, Chun‐Chao Chang, Wei‐Lun Chang, Jaw‐Town Lin

**Affiliations:** ^1^ Departments of Institute of Clinical Medicine and Internal MedicineNational Cheng Kung University HospitalCollege of MedicineNational Cheng Kung UniversityTainanTaiwan; ^2^ Department of Internal MedicineTainan HospitalMinistry of Health and WelfareTainanTaiwan; ^3^ Department of Internal MedicineNational Taiwan University HospitalTaipeiTaiwan; ^4^ Gastroenterology Endoscopy CenterChang Gung Memorial HospitalLinkoTaiwan; ^5^ Department of Internal MedicineTaipei Veterans General HospitalTaipeiTaiwan; ^6^ Department of Internal MedicinePrivate Kaohsiung Medical University HospitalKaohsiungTaiwan; ^7^ Department of Internal MedicineTaichung Veterans General HospitalTaichungTaiwan; ^8^ Department of Internal MedicineKaohsiung Veterans General HospitalKaohsiungTaiwan; ^9^ Department of Internal MedicinePrivate Taipei Medical University HospitalTaipeiTaiwan; ^10^ School of MedicineFu Jen Catholic UniversityNew Taipei CityTaiwan

**Keywords:** consensus, endoscopy, eradication, gastric cancer, gastric precancerous lesion, *Helicobacter pylori*, level of evidence, peptic ulcer, proton‐pump inhibitor

## Abstract

**Background:**

Previous international consensus statements provided general policies for the management of *Helicobacter pylori* infection. However, there are geographic differences in the prevalence and antimicrobial resistance of *H. pylori*, and in the availability of medications and endoscopy. Thus, nationwide or regional consensus statements are needed to improve control of *H. pylori* infection and gastric cancer.

**Materials and Methods:**

This consensus statement for management of *H. pylori* in Taiwan has three major sections: (1) optimal diagnosis and indications; (2) current treatment strategies; and (3) screening‐to‐treat and surveillance for control of gastric cancer. The literature review emphasized recent data for development of draft statements and determination of levels of evidence. Twenty‐five Taiwan experts conducted a consensus conference, by a modified Delphi process, to modify the draft statements. Consensus, defined as an agreement of least 80% of the experts, and recommendation grade were determined by anonymous voting.

**Results:**

There were 24 consensus statements. Section 1 has seven statements on recommendations for the diagnosis and indications for treatment of *H. pylori* infection. Section 2 has 10 statements that provide an updated treatment algorithm for first‐line, second‐line, and third‐line regimens. Section 3 has seven statements regarding *H. pylori* eradication for reducing the risk of gastric cancer, with a cost‐benefit analysis. After *H. pylori* eradication, the consensus highlights the use of endoscopic surveillance and/or chemoprevention to further reduce the burden of gastric cancer.

**Conclusions:**

This consensus statement has updated recommendations for improving the clinical management of *H. pylori* infection in areas such as Taiwan, which have high prevalence of *H. pylori* infection and gastric cancer.

**Abbreviations**
CI: confidence interval
COI: conflict of interest
COX‐2: cyclooxygenase‐2
GERD: gastroesophageal reflux disease
HR: hazard ratio
MALToma: mucosa‐associated lymphoid tissue lymphoma
NA: not applicable
NHIRD: National Health Insurance Research Database
NSAIDs: nonsteroid anti‐inflammatory drugs
OLGA: operative link for gastric atrophy assessment
OLGIM: Operative Link on Gastric Intestinal Metaplasia Assessment
PPI: proton‐pump inhibitor
RCTs: randomized controlled trials
RR: relative risk
SAT: stool antigen test
UBT: urea breath test
WHO: World Health Organization

## 1 Introduction

Marshall and Warren discovered *Helicobacter pylori* in the biopsies of patients with gastritis and peptic ulcers in 1983,^1^ and the World Health Organization classified *H. pylori* infection as a Group I carcinogen for gastric cancer in 1994. Since then, *H. pylori* eradication has been an established method for control of peptic ulcers.^2^, ^3^, ^4^, ^5^, ^6^, ^7^ After Marshall and Warren received the Nobel Prize in 2005, basic and clinical research worldwide focused on the effects of unresolved *H. pylori* infections.^4^ Basic research has focused on the relationship of specific host‐bacterial interactions with clinical outcome, in efforts to develop effective preventative or therapeutic vaccines. Clinical studies have focused on three general topics: (1) better clinical management of *H. pylori* infection by increasing the accuracy of diagnosis and defining the exact indications for treatment; (2) more successful eradication of *H. pylori* infection by development of more effective treatment strategies; and (3) performance of large‐scale screening‐to‐treat and surveillance of *H. pylori* infections to improve control of gastric cancer.

The European *Helicobacter* Study Group has delivered serial editions of the Maastricht Consensus to update guidelines for the management of *H. pylori* infection.^5^, ^6^ The Asia‐Pacific Gastric Cancer Consensus Conference has provided the guidelines on gastric cancer prevention.^7^ The Toronto Consensus recently reported its recommendations for treatment of *H. pylori* infection.^8^ These consensus statements can have high impact on the clinical management of *H. pylori* infection worldwide. Nevertheless, there are geographic differences in the effect of *H. pylori* infection on the risk of gastric cancer, the prevalence of antimicrobial resistance to *H. pylori*, and the availability of medications and endoscopy facilities. Thus, consensus statements must be revised due to changes over time and differences among populations, so that the optimal strategy is used to control peptic ulcer and gastric cancer.

In Taiwan, endoscopy and related therapeutic modalities are readily available on a nationwide scale. The National Health Insurance (NHI) program, which covers more than 99% of the population of Taiwan, provides full support for medications used to eradicate *H. pylori* and endoscopy for pretreatment screening and post‐treatment surveillance. Nonetheless, gastric cancer remains a major cancer in Taiwan.^9^ Thus, we developed this consensus statement to improve the clinical management of *H. pylori* infection in Taiwan and to provide updated treatment strategy recommendations that improve the success of eradication. Moreover, the availability of the Taiwan NHI database allows us to develop a consensus statement with strong validity, because it is based on a nationwide cohort with data on the benefit of screening‐to‐treat for *H. pylori* infection and the potential need for endoscopic surveillance of gastric precancerous lesions after *H. pylori* eradication to reduce the risk of gastric cancer. Our recommendations for the population of Taiwan, which has a high prevalence of *H. pylori* infection and gastric cancer, may also be helpful for other regions in Asia in their efforts to improve *H. pylori* eradication to reduce the risk of gastric cancer.

## 2 Methods

### 2.1 Scope, setting, and structure for preparation of the consensus statement

The steering committee that established the expert consensus statement for clinical management of test‐to‐treat screening and surveillance of *H. pylori* infection and improved control of peptic ulcer and gastric cancer in Taiwan was initiated by JT Lin, chaired by BS Sheu, and co‐chaired by MS Wu. There were also 10 other opinion leaders from the Gastroenterological Society of Taiwan (Chiu CT, Lo CJ, Wu DC, Liou CM, Wu CY, Cheng HC, Lee YC, Hsu PI, Chang CC, and Chang WL). The 13 members of the steering committee defined the scope of the sections of the consensus statement, searched for and reviewed the relevant literature, formulated the draft statements, and defined the level of statement evidence.

### 2.2 Search and review of literature to initiate draft statements, and grading of evidence level

The literature was searched using MEDLINE, Embase, the Cochrane Central Register of Controlled Trials, and the ISI Web of Knowledge. There were also manual searches of the bibliographies of key articles and proceedings of abstracts of major gastroenterology conferences over the past 20 years (January 1996 to March 2016). The key words used in the search were the following: *Helicobacter pylori*, peptic ulcer, gastric cancer, gastric B‐cell lymphoma, gastric atrophy, intestinal metaplasia, eradication therapy, antimicrobial resistance, cost‐effectiveness, and Taiwan. The literature review focused on unique findings from Taiwan, for comparisons with major articles from throughout the world.

The steering committee summarized the findings in three sections of this consensus statement: (1) optimal diagnostic approach and indications for treatment for *H. pylori* infection; (2) current treatment strategies used for first‐line, second‐line, and third‐line eradication of *H. pylori*; and (3) cost‐effectiveness of screening‐to‐treat and surveillance for *H. pylori* infection to improve control of gastric cancer. In the last section, the consensus statement introduces current evidence from Taiwan, based on analysis of the NHI Registry Database (NHIRD), to assess the role of *H. pylori* eradication on reducing the risk of gastric cancer with a cost‐benefit analysis. Based on review of the literature, the draft statements of this consensus were established by the section leader(s). For each statement, the level of evidence was defined according to the modified grading system of the Oxford Centre for Evidence‐Based Medicine Levels of Evidence (March, 2009).^10^ In 2015, the steering committee meeting refined the draft statements for each section at meetings during March (Tainan), June (Taipei), and September (Kaohsiung).

### 2.3 Expert group process to achieve agreement of statement and recommendation grade

The expert group had 25 experts, 13 members of the steering committee and 12 members who accepted invitations. These experts were chosen based on their expertise and contributions to the published literature. The draft statements from the four sessions were sent to all experts, together with pertinent literature, before the consensus meeting on March 5‐6, 2016, in Tainan. During this 2‐day consensus meeting, each draft statement from the four sessions, with supporting evidence from the keynote literature summary by the steering committee, was presented sequentially as optimal diagnosis and indications for treatment; current treatment strategies for *H. pylori* infection; and screen‐to‐treat and surveillance of *H. pylori* infection to control gastric cancer.

Based on a modified Delphi process with two separate iterations, all participants voted anonymously for the first round of statements, and modified these statements by discussion. Then, there was a second round of voting until a consensus was reached (agreement by at least 80% of the expert members). Statements were rejected if the agreement was <80%.

The experts also discussed the level of evidence suggested by the steering committee and graded the recommendations by voting for each statement. The recommendation grade ranged from A to D, as in our previous consensus statement.^10^ Each grade was defined by the most votes. Table 1 shows the entire statements, with levels of scientific evidence and grades of recommendation. The conferences in which this work was performed were underwritten by unrestricted grants from the Gastroenterological Society of Taiwan and Ministry of Health and Welfare, Taiwan (R.O.C.). Before voting, all experts provided written disclosures of financial conflicts of interest in the 3 years before the meetings.

**Table 1:** Summary of the consensus statements for the management of *H. pylori* infection, with levels of scientific evidence and grades of recommendation

Consensus statements	Level of evidence	Grade of recommendation
I. Optimal *H. pylori* diagnosis & indications for treatment		
I‐1a. Endoscopic gastric biopsy with histological analysis and the rapid urease test can accurately detect *H. pylori* infection	2b	A
I‐1b. The urea breath test (UBT) and stool antigen test (SAT) can noninvasively and accurately detect *H. pylori* infection	2a	A
I‐1c. The UBT is preferred for detection of *H. pylori* infection in patients with bleeding peptic ulcers	2a	A
I‐2. Confirmation of *H. pylori* eradication requires retesting at least 4 weeks after cessation of antibiotics, and 2 weeks after cessation of a proton‐pump inhibitor (PPI)	2b	A
I‐3a. The UBT or SAT is effective for mass screening of active *H. pylori* infection	2b	A
I‐3b. When a fecal immunochemical test is performed for colon cancer screening, a simultaneous SAT may help to detect *H. pylori* infection	2b	B
I‐3c. A serological test, although less specific, is effective to assess the prevalence of *H. pylori* in epidemiological studies	2b	A
I‐4a. *H. pylori* eradication decreases the risk of peptic ulcer disease	1b	A
I‐4b. *H. pylori* eradication reduces peptic ulcer recurrence and recurrent bleeding	1a	A
I‐5. *H. pylori* eradication is the first‐line treatment for early‐stage gastric MALToma	2a	A
I‐6a. *H. pylori* eradication improves symptom control in *H. pylori*‐infected patients who have dyspepsia	1a	A
I‐6b. Asian and Western populations differ in the risk of GERD after *H. pylori* eradication	1a	NA
I‐7a. *H. pylori* eradication reduces the risk of peptic ulcer in NSAID‐naïve users	1a	A
I‐7b. After *H. pylori* eradication in patients with NSAID‐induced ulcer bleeding, a PPI plus a COX‐2 inhibitor is best for prevention of recurrent ulcer bleeding	1b	A
I‐8. After *H. pylori* eradication, long‐term use of a PPI is needed to prevent recurrent ulcer or ulcer bleeding in long‐term users of antiplatelet therapies	1b	A
II. Current treatment strategies for *H. pylori* infection		
II‐1a. Clarithromycin‐based triple therapy is the best first‐line regimen for *H. pylori* infection in geographic regions with the prevalence of primary clarithromycin resistance below 15%	1a	A
II‐1b. Bismuth quadruple therapy is a suitable alternative to triple therapy in geographic regions with the prevalence of primary clarithromycin resistance below 15%	1a	A
II‐1c. Bismuth quadruple therapy provides a better eradication rate than triple therapy in geographic regions with the prevalence of primary clarithromycin resistance above 15%	1a	A
II‐2. Extending the duration of clarithromycin triple therapy from 7 or 10 to 14 days improves the eradication rate of *H. pylori*	1a	A
II‐3. Hybrid, sequential, and concomitant therapies are superior to clarithromycin‐triple therapy of the same duration for the eradication of *H. pylori*	1a	A
II‐4. A higher PPI dose increases the eradication rate of *H. pylori* from triple therapy with clarithromycin & amoxicillin as a first‐line treatment	1a	A
II‐5. Bismuth quadruple therapy is effective in patients with penicillin allergies, and triple therapy with clarithromycin & metronidazole is an effective alternative if bismuth is not available	2b	A
II‐6. Supplementation with certain probiotics, such as Lactobacillus or *Saccharomyces boulardii*, increases the eradication rate and reduces the adverse effects of antibiotic treatment	1a	B
II‐7. Levofloxacin triple therapy given for 10‐14 days is more effective and better tolerated than bismuth quadruple therapy as a second‐line treatment	1a	A
II‐8. Clarithromycin and levofloxacin should not be reused in rescue therapy unless the *H. pylori* isolate has proven susceptibility to these antibiotics	2a	A
II‐9. Therapy guided by susceptibility‐testing is recommended for patients who fail two or more eradication therapies	4	B
II‐10. For patients treated with a PPI‐based triple therapy, those with the CYP2C19 loss‐of‐function variant have a higher eradication rate	2a	NA
III. Screening‐to‐treat and surveillance for gastric cancer control		
III‐1. *H. pylori* eradication reduces the incidence of gastric cancer and progression of premalignant gastric lesions	1a	A
III‐2. *H. pylori* eradication reduces the risk of metachronous gastric cancer after tumor resection	1b	A
III‐3. After *H. pylori* eradication, NSAIDs may provide chemoprevention by halting the progression of premalignant lesions, and thereby reduce the risk of gastric cancer; however, their adverse effects should be considered	2b	B
III‐4. A screen‐to‐treat approach for management of *H. pylori* infection should cost‐effectively reduce the incidence of gastric cancer in intermediate‐ and high‐risk populations	2a	B
III‐5. After *H. pylori* eradication, subjects with gastric premalignant lesions still have increased risk of gastric cancer, and thus need endoscopic surveillance at scheduled intervals stratified by the OLGA/OLGIM system	2b	A
III‐6. Patients with high‐grade gastric intraepithelial neoplasia (dysplasia) should receive endoscopic or surgical resection	2b	A
III‐7. Serum pepsinogens, anti‐*H. pylori* antibodies, and certain demographic characteristics are useful in identifying subjects with high risk for gastric cancer	2a	B

The level of evidence and grade of recommendation were defined according to modified grading of the Oxford Centre for Evidence‐Based Medicine Levels of Evidence.

## 3 Consensus Statements

### 3.1 Section I: Optimal diagnosis & indications for treatment of *H. pylori* infection

Statement I‐1a: Endoscopic gastric biopsy with histological analysis and the rapid urease test can accurately detect *H. pylori* infection. ( Evidence level: 2b, Agreement: 100%, Recommendation: A)

Statement I‐1b: The urea breath test (UBT) and stool antigen test (SAT) can non‐invasively and accurately detect *H. pylori* infection. (Evidence level: 2a, Agreement: 100%, Recommendation: A)

Statement I‐1c: The UBT is preferred for detection of *H. pylori* infection in patients with bleeding peptic ulcers. (Evidence level: 2a, Agreement: 96%, Recommendation: A)

Clinicians can detect *H. pylori* infections using invasive or noninvasive procedures. During upper gastroduodenal endoscopy, the rapid urease test is a simple, rapid, and highly reliable test for *H. pylori* infection.^11^, ^12^ Histological analysis of the gastric biopsy obtained during endoscopy can also provide a highly accurate detection of *H. pylori* infection.^13^ A meta‐analysis confirmed that the UBT is highly accurate in detection of *H. pylori* infection in patients with dyspepsia.^14^ Another meta‐analysis confirmed that the SAT, which uses a mixture of monoclonal antibodies against *H. pylori*, is an accurate and noninvasive method for detection of *H. pylori*.^15^ The UBT is preferred for patients with bleeding peptic ulcers, because the rapid urease test tends to produce false‐negative results due to the presence of gastric blood.^16^ Nevertheless, for patients with gastrectomy and uremia, the UBT has reduced accuracy in detection of *H. pylori* infection,^17^, ^18^ so the SAT can also be considered.^19^, ^20^

Statement I‐2: Confirmation of *H. pylori* eradication requires retesting at least 4 weeks after cessation of antibiotics, and 2 weeks after cessation of a proton pump inhibitor (PPI). (Evidence level: 2b, Agreement: 96%, Recommendation: A)

To confirm *H. pylori* eradication, retesting, either with a noninvasive or invasive test, is needed. To increase the test accuracy, this test should be performed at least 4 weeks after cessation of antibiotics and at least 2 weeks after cessation of a PPI.^21^, ^22^, ^23^, ^24^

Statement I‐3a: The UBT or SAT is effective for mass screening of active *H. pylori* infection. (Evidence level: 2b, Agreement: 92%, Recommendation: A)

Statement I‐3b: When a fecal immunochemical test is performed for colon cancer screening, a simultaneous SAT may help to detect *H. pylori* infection. (Evidence level: 2b, Agreement: 92%; Recommendation: B)

Statement I‐3c: A serological test, although less specific, is effective to assess the prevalence of *H. pylori* in epidemiological studies. (Evidence level: 2b, Agreement: 92%, Recommendation: A)

The UBT is a simple method used to screen for active *H. pylori* infection in the community.^25^, ^26^ The SAT is also feasible and has acceptable sensitivity (88.0%), excellent specificity (100%), and a high participation rate (77.3%) in screening for *H. pylori* infection in large‐scale community studies.^27^ For subjects receiving colon cancer screening with the fecal immunochemical test, the concurrent use of the SAT for detection of *H. pylori* may provide additional benefit.^28^ A less expensive serological test can be used to assess the *H. pylori* prevalence in epidemiological studies, but it has a lower specificity and a high risk for false‐positive results.^29^, ^30^

Statement I‐4a: *H. pylori* eradication decreases the risk of peptic ulcer disease. (Evidence level: 1b, Agreement: 96%, Recommendation: A)

Statement I‐4b: *H. pylori* eradication reduces peptic ulcer recurrence and recurrent bleeding. (Evidence level: 1a, Agreement: 100%, Recommendation: A)

*H. pylori* eradication can prevent the subsequent development of peptic ulcers in patients with nonulcer dyspepsia.^31^ A 5‐year longitudinal follow‐up study confirmed that mass eradication of *H. pylori* infection in 4121 community participants reduced the progression of premalignant gastric lesions and the occurrence of peptic ulcer disease.^26^

For patients with persistent *H. pylori* infections, peptic ulcers can recur after healing. A meta‐analysis of 56 randomized controlled trials (RCTs) showed that *H. pylori* eradication reduced the relative risk (RR) of recurrent gastric ulcer (RR=0.20; 95% confidence interval [CI]: 0.15‐0.26) and duodenal ulcer (RR=0.29; 95% CI: 0.20‐0.42).^32^ Another meta‐analysis reported that *H. pylori* eradication significantly decreased the recurrence of peptic ulcer bleeding.^33^

Statement I‐5: *H. pylori* eradication is the first‐line treatment for early‐stage gastric MALToma. (Evidence level: 2a, Agreement: 92%, Recommendation: A)

*Helicobacter pylori* infection has a key pathogenic role in marginal zone B‐cell mucosa‐associated lymphoid tissue lymphoma (MALToma).^22^, ^34^, ^35^, ^36^, ^37^ A retrospective, long‐term study indicated that anti‐*H. pylori* therapy led to 78% complete or partial remission of localized gastric MALToma.^34^ Prospective trials in Taiwan reported that *H. pylori* eradication led to complete remission in 80% of patients with early‐stage gastric MALToma^35^, ^36^ and 64% of patients with diffuse large B‐cell high‐grade MALToma.^37^ In addition, the 5‐year recurrence rate was 13% patients with for those with low‐grade cancer, and 0% for those with high‐grade cancer.^37^ *H. pylori* eradication is thus an effective first‐line treatment for early‐stage (*i.e,* confined to stomach) *H. pylori*‐positive gastric MALToma.

Statement I‐6a: *H. pylori* eradication improves symptom control in *H. pylori*‐infected patients who have dyspepsia. (Evidence level: 1a, Agreement: 84%, Recommendation: A)

Statement I‐6b: Asian and Western populations differ in the risk of GERD after *H. pylori* eradication. (Evidence level: 1a, Agreement:100%, Recommendation: NA)

The Kyoto consensus emphasizes the etiology‐based investigation of gastritis and recommends classification of gastritis as “*H. pylori*‐induced” or as “*H. pylori*‐negative” (or idiopathic).^38^ In practice, if there is sustained symptom relief after antibiotic treatment, the disease is considered *H. pylori*‐associated dyspepsia; if the symptoms do not resolve, the disease is considered functional dyspepsia. A Cochrane meta‐analysis of the effect of *H. pylori* eradication on dyspepsia symptoms examined 3566 patients, and the results indicated a 10% lower RR of symptoms after eradication during the long‐term follow‐up.^39^ In primary care clinics, a RCT reported that *H. pylori* eradication provided better symptom control in patients with *H. pylori* infections.^40^ In addition to symptom control, the rate of subsequent ulcer development is also lower after eradication.^31^

The prevalence of gastroesophageal reflux disease (GERD) is increasing worldwide, including Taiwan.^41^ The impact of *H. pylori* eradication on the severity of GERD remains controversial. *H. pylori* infection may lead to gastric inflammation and thus may trigger stronger acid secretion in patients with antrum‐predominant gastritis. Thus, *H. pylori* eradication may better control acid secretion without triggering pre‐existing GERD or causing new‐onset GERD. In contrast, the low gastric acid secretion in patients with *H. pylori*‐associated corpus‐predominant or pan‐gastritis might be restored after *H. pylori* eradication, and this might lead to aggravation of pre‐existing symptoms of GERD. Two meta‐analyses of studies that mainly examined Western populations showed no significant increase in GERD after *H. pylori* eradication.^42^, ^43^ However, a recent meta‐analysis of 6158 patients showed that *H. pylori* eradication led to a higher risk of new‐onset GERD in Asians (RR: 4.53, 95% CI: 1.66‐12.36), but not in Westerners (RR: 1.22, 95% CI: 0.91‐1.63).^44^ The increased body mass index in Asian people after *H. pylori* eradication is a possible trigger for new‐onset GERD.^45^

Statement I‐7a: *H. pylori* eradication reduces the risk of peptic ulcer in NSAID‐naïve users. (Evidence level: 1a, Agreement: 96%, Recommendation: A)

Statement I‐7b: After *H. pylori* eradication in patients with NSAID‐induced ulcer bleeding, a PPI plus a COX‐2 inhibitor is best for prevention of recurrent ulcer bleeding. (Evidence level: 1b, Agreement: 96%, Recommendation: A)

*Helicobacter pylori* infection and use of nonsteroid anti‐inflammatory drugs (NSAIDs) are independent risk factors that additively increase the risk of peptic ulcer.^46^ In NSAID‐naive users, *H. pylori* eradication can effectively prevent NSAID‐related ulcers and ulcer bleeding,^47^, ^48^ but this benefit is limited to long‐term users.^49^, ^50^ Among patients with NSAID‐related peptic ulcer bleeding, long‐term use of a PPI plus a cycloxygenase‐2 (COX‐2) inhibitor achieved better control of recurrent ulcer bleeding than *H. pylori* eradication or a COX‐2 inhibitor alone.^51^ *H. pylori* eradication alone may be insufficient to control the long‐term recurrent bleeding of NSAID‐related ulcers. At present, the optimal strategy for prevention of recurrent ulcer bleeding in NSAID users after *H. pylori* eradication seems to be use of a PPI plus a COX‐2 inhibitor.

Statement I‐8: After *H. pylori* eradication, long‐term use of a PPI is needed to prevent recurrent ulcer or ulcer bleeding in long‐term users of anti‐platelet therapies. (Evidence level: 1b, Agreement: 100%, Recommendation: A)

An observational study in Hong Kong showed that the long‐term incidence of recurrent ulcer bleeding was low in aspirin users after *H. pylori* eradication.^52^ However, a RCT showed that maintenance PPI therapy for 12 months after *H. pylori* eradication further reduced the risk of recurrent ulcer bleeding in low‐dose aspirin users who experienced aspirin‐associated ulcer bleeding, compared with those receiving *H. pylori* eradication alone.^53^ Another RCT showed that the risk of rebleeding was lower in patients treated with a PPI plus aspirin than those treated with clopidogrel alone.^54^ Therefore, we recommend that long‐term antiplatelet users receive maintenance PPI therapy to reduce the risk of recurrent ulcer bleeding after *H. pylori* eradication.^55^

### 3.2 Section II: Current treatment strategies for *H. pylori* infection

**Figure 1:**
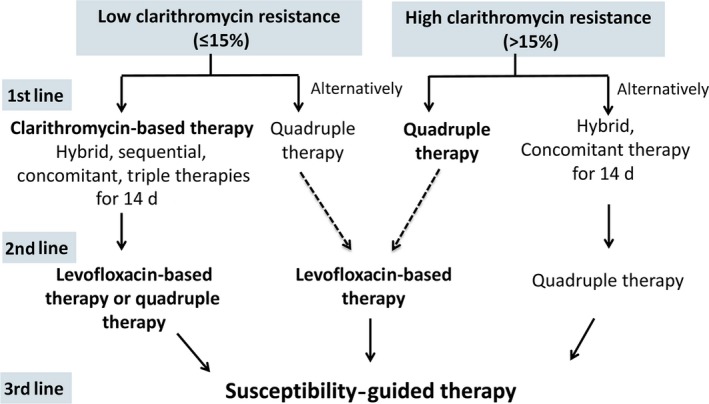
The algorithm for the recommended treatment of *H. pylori* infection **(Agreement: 100%)**. In areas with low clarithromycin resistance (≤15%), a 14‐d clarithromycin‐based therapy (hybrid, sequential, concomitant, or triple therapy) is the treatment of choice; a levofloxacin‐based therapy and quadruple therapy are effective second‐line (rescue) therapies. A 10‐ to 14‐d bismuth quadruple therapy is a suitable alternative first‐line therapy; a levofloxacin‐based therapy is suitable as a second‐line (rescue) therapy. The dashed lines in the figure indicate the lack of high level of evidence. In areas with high clarithromycin resistance (>15%), a 10‐ to 14‐d bismuth quadruple therapy is effective; a levofloxacin‐based is suitable as a second‐line (rescue) therapy; a 14‐d hybrid or concomitant therapy is an alternative first‐line therapy; a bismuth quadruple therapy is suitable as a second‐line (rescue) therapy. Drug choice guided by susceptibility testing should be used for patients who fail two eradication therapies

The *H. pylori* eradication rate following clarithromycin‐based triple therapy has fallen below 80% due to emerging antibiotic resistance worldwide.^56^, ^57^ Several strategies can improve the eradication rate of the first‐line treatment (Table**** 2)^5^, ^6^, ^7^, ^8^, ^58^, ^59^, ^60^, ^61^, ^62^, ^63^, ^64^, ^65^: selection of a clarithromycin‐free regimen^60^, ^61^; use of clarithromycin, but expansion to a four‐drug combination treatment course as a sequential, concomitant, or hybrid therapy^62^, ^63^, ^64^, ^65^; and extension of the triple therapy to 14 days and use of a higher PPI dosage.^66^, ^67^, ^68^ We also address the current optimal therapy for patients with penicillin allergies and suggest rational rescue therapies as second‐line and third‐line treatments (Figure 1).

**Table 2:** The effective first‐line *H. pylori* eradication regimens alternative to the clarithromycin‐based triple therapy

Concomitant therapy for 7‐14 d	
Proton‐pump inhibitor, 20‐40 mg (depending on drug), twice daily	
Amoxicillin, 1 g, twice daily	
Metronidazole, 500 mg, twice daily	
Clarithromycin, 500 mg, twice daily	
Sequential therapy for 10‐14 d	
Proton‐pump inhibitor, 20‐40 mg (depending on drug), twice daily	
Days 1‐5 (or 1‐7) Amoxicillin, 1 g, twice daily	Days 6‐10 (or 8‐14)Metronidazole, 500 mg, twice daily
	Clarithromycin, 500 mg, twice daily
Hybrid therapy for 10 or 14 d	
Proton‐pump inhibitor, 20‐40 mg (depending on drug), twice daily	
Days 1‐5 (or 1‐7) Amoxicillin, 1 g, twice daily	Days 6‐10 (or 8‐14) Amoxicillin, 1 g, twice daily
	Metronidazole, 500 mg, twice daily
	Clarithromycin, 500 mg, twice daily
Quadruple therapy for 7, 10, or 14 d	
Proton‐pump inhibitor, 20‐40 mg (depending on drug), twice daily	
Colloidal bismuth subcitrate, 300 mg, four times daily	
Metronidazole, 500 mg, three times daily	
Tetracycline, 500 mg, four times daily	
Levofloxacin triple therapy for 7, 10, or 14 d	
Proton‐pump inhibitor, 20‐40 mg (depending on drug), twice daily	
Amoxicillin, 1 g, twice daily	
Levofloxacin, 500 mg, once daily (or 250 mg, twice daily)	

Statement II‐1a: Clarithromycin‐based triple therapy is the best first‐line regimen for *H. pylori* infection in geographic regions with the prevalence of primary clarithromycin resistance below 15%. (Evidence level: 1a, Agreement: 100%, Recommendation: A)

Statement II‐1b: Bismuth quadruple therapy is a suitable alternative to triple therapy in geographic regions with prevalence of primary clarithromycin resistance below 15%. (Evidence level: 1a, Agreement: 92%, Recommendation: A)

Statement II‐1c: Bismuth quadruple therapy provides a better eradication rate than triple therapy in geographic regions with prevalence of primary clarithromycin resistance above 15%. (Evidence level: 1a, Agreement: 92%, Recommendation: A)

The prevalence of clarithromycin resistance is lower than 15% in most regions of Taiwan.^63^, ^69^ A RCT with a crossover design confirmed clarithromycin triple therapy was more effective than levofloxacin triple therapy as a first‐line treatment for *H. pylori* infection in Taiwan.^69^ Moreover, this study also showed that use of clarithromycin triple therapy as a first‐line treatment and levofloxacin triple therapy as a second‐line treatment achieved a higher overall eradication rate than the reverse sequence.^69^ In agreement, a large‐scale meta‐analysis showed that levofloxacin triple therapy was not superior to clarithromycin triple therapy as a first‐line treatment.^61^ Accordingly, clarithromycin triple therapy remains the first‐line treatment in Taiwan and in other countries in which the clarithromycin resistance rate is below 15%.

Another meta‐analysis of RCTs showed that bismuth quadruple therapy was not superior to clarithromycin triple therapy when these regimens were given for the same duration.^70^, ^71^ However, a prolonged bismuth quadruple therapy (10 days) could be superior to the standard 7‐day triple clarithromycin therapy in regions with clarithromycin resistance rates above 15%.^60^, ^72^ Liou et al. recently showed that a 10‐day bismuth quadruple therapy was superior to 14‐day triple therapy in Taiwan.^73^ Therefore, bismuth quadruple therapy is an alternative first‐line regimen for geographic areas with low clarithromycin resistance, and is the treatment of choice in regions with high clarithromycin resistance (15% or more).

Statement II‐2: Extending the duration of clarithromycin triple therapy from 7 or 10 to 14 days improves the eradication rate of *H. pylori*. (Evidence level: 1a, Agreement: 100%, Recommendation: A)

Two meta‐analysis studies confirmed that extending the treatment duration of clarithromycin triple therapy to 14 days achieved a higher eradication rate than the standard 7‐ or 10‐day treatment.^67^, ^74^ Moreover, a 14‐day levofloxacin triple therapy is superior to a 7‐day clarithromycin triple therapy for *H. pylori* eradication.^75^ Therefore, we recommend use of a 14‐day clarithromycin triple therapy to increase the eradication rate.

Statement II‐3: Hybrid, sequential, and concomitant therapies are superior to clarithromycin‐triple therapy of the same duration for the eradication of *H. pylori*. (Evidence level: 1a, Agreement: 100%, Recommendation: A)

Several randomized trials and meta‐analyses showed that nonbismuth four‐drug therapies containing clarithromycin were superior to triple therapy for *H. pylori* eradication when given for 7 or 10 days.^62^ A study in Taiwan reported that a 7‐day concomitant therapy was superior to a 7‐day triple therapy.^76^ A randomized trial also showed that 14‐day sequential therapy (but not a 10‐day sequential therapy) was superior to 14‐day triple therapy.^63^, ^77^ Additional research indicated that hybrid and reverse hybrid therapies were highly effective first‐line treatments.^66^, ^78^ Several trials showed that 14‐day concomitant or hybrid therapies were less affected by clarithromycin resistance and are effective alternatives to bismuth quadruple therapy in regions with high clarithromycin resistance.^62^, ^66^, ^77^, ^78^, ^79^ Another randomized trial showed that a 10‐day bismuth quadruple therapy (but not a 10‐day concomitant therapy) was superior to a 14‐day triple therapy.^73^ Taken together, these results support the use of nonbismuth quadruple therapies rather than triple therapy when these are given for the same duration. We therefore recommend that nonbismuth quadruple therapy be given for 14 days, whereas bismuth quadruple therapy may be given for 10‐14 days. Further randomized trials are needed to compare the efficacy of nonbismuth quadruple therapies with bismuth quadruple therapy.

Statement II‐4: A higher PPI dose increases the eradication rate of *H. pylori* from triple therapy with clarithromycin & amoxicillin as a first‐line treatment. (Evidence level: 1a, Agreement: 100%, Recommendation: A)

The standard PPI dose (given twice daily) for *H. pylori* eradication is 20 mg omeprazole, 30 mg lansoprazole, 20 mg esomeprazole, 40 mg pantoprazole, and 20 mg rabeprazole. Sheu et al. studied patients in Taiwan and found that esomeprazole, at 40 mg twice daily, improved the *H. pylori* eradication rate of triple therapy, even for individuals with the cytochrome 2C19 genotype rapid metabolizers.^80^ Large‐scale meta‐analyses supported the benefits of using a higher PPI dose to improve the eradication rate of standard triple therapy.^68^, ^81^ No studies have yet examined the efficacy of a higher PPI dose on the efficacy of bismuth or nonbismuth quadruple therapy.

Statement II‐5: Bismuth quadruple therapy is effective in patients with penicillin allergies, and triple therapy with clarithromycin & metronidazole is an effective alternative if bismuth is not available. (Evidence level: 2b, Agreement: 100%, Recommendation: A)

Regimens without amoxicillin, such as bismuth quadruple therapy and triple therapy with metronidazole and clarithromycin, may be used for patients with penicillin allergies.^58^, ^59^ A recent trial showed that the bismuth quadruple therapy was more effective than triple therapy with metronidazole and clarithromycin.^82^

Statement II‐6: Supplementation with certain probiotics, such as Lactobacillus or Saccharomyces boulardii, increases the eradication rate and reduces the adverse effects of antibiotic treatment. (Evidence level: 1a, Agreement: 92%, Recommendation: B)

Two meta‐analyses of RCTs showed that supplementation with probiotics increased the eradication rate and reduced the adverse effects of triple therapy.^83^, ^84^ However, only *Lactobacillus acidophilus*,* L. casei*,* L. gasseri*, and *Bifidobacterium infantis* were effective in a subgroup analysis.^28^ Although the Toronto Consensus did not recommend supplementation with probiotics,^8^ studies in Taiwan showed that supplementation with *Lactobacillus‐* and *Bifidobacterium*‐containing yogurt increased the efficacy of triple therapy and quadruple therapy when given as first‐line and second‐line treatments, respectively.^85^, ^86^ Therefore, we recommend supplementation with probiotics to increase the eradication rate and reduce adverse effects. This recommendation is consistent with the Maastricht V statement, that only certain probiotics are effective to reduce side effects caused by *H. pylori* eradication therapies. Specific strains should be chosen only if they have demonstrated clinical efficacy.^7^

Statement II‐7: Levofloxacin triple therapy given for 10‐14 days is more effective and better tolerated than bismuth quadruple therapy as a second‐line treatment. (Evidence level: 1a, Agreement: 96%, Recommendation: A)

The efficacies of 7‐day levofloxacin triple therapy and 7‐day quadruple therapy were similar when used as second‐line treatments of patients in Taiwan.^87^ Triple therapy with levofloxacin (500 mg, once daily) can be sufficient as a second‐line therapy.^88^ A meta‐analysis of RCTs showed that 10‐14 days of a levofloxacin triple therapy was more effective and better tolerated than bismuth quadruple therapy as a second‐line treatment.^89^ It is noteworthy that the efficacy of levofloxacin triple therapy as a second‐line treatment has fallen below 80% in recent years,^90^ although a recent nationwide study in Taiwan indicated that levofloxacin sequential therapy remained effective.^91^

Statement II‐8: Clarithromycin and levofloxacin should not be reused in rescue therapy unless the *H. pylori* isolate has proven susceptibility to these antibiotics. (Evidence level: 2a, Agreement: 96%, Recommendation: A)

The secondary resistance to clarithromycin and levofloxacin is high in patients who failed regimens containing these antibiotics.^57^, ^92^ Accordingly, the reuse of clarithromycin and levofloxacin empirically should be avoided.

Statement II‐9: Therapy guided by susceptibility‐testing is recommended for patients who fail two or more eradication therapies. (Evidence level: 4, Agreement: 92%, Recommendation: B)

Susceptibility‐guided therapy is more effective than empirical therapy as a first‐line treatment.^93^ More specifically, two case series showed the eradication rate of susceptibility‐guided therapy ranged from 36% to 91%.^94^, ^95^ However, susceptibility testing is expensive and not widely available. Mutations in 23S rRNA correlate positively with clarithromycin resistance and negatively with the efficacy of clarithromycin regimens, and mutations in gyrase A correlate positively with levofloxacin resistance and negatively with the efficacy of levofloxacin regimens.^96^ In Taiwan, a pilot study showed that genotype resistance‐guided therapy achieved an 80% eradication rate as a third‐line treatment.^97^ Further trials are needed to validate whether such tailored therapies are superior to empirical therapy.

Statement II‐10: For patients treated with a PPI‐based triple therapy, those with the CYP2C19 loss‐of‐function variant have a higher eradication rate. (Evidence level: 2a, Agreement: 100%, Recommendation: NA)

Most PPIs are metabolized by the liver enzyme cytochrome P450 2C19 (CYP2C19). Subjects with the CYP2C19 loss‐of‐function variant (poor metabolizers) therefore have higher serum concentrations of PPIs during treatment than those without this variant (extensive metabolizers).^80^ A meta‐analysis showed that *H. pylori‐*infected patients with the loss‐of‐function variant experienced a higher eradication rate of *H. pylori* from a PPI‐based triple therapy than homozygous and heterozygous extensive metabolizers.^98^

### 3.3 Section III: Screening‐to‐treat and surveillance for control of gastric cancer

Gastric cancer is the fifth most common human cancer and the third most common cause of cancer‐related deaths worldwide.^99^ Eradication of *H. pylori* infection has the potential to decrease the risk of gastric cancer.^100^ Therefore, a screening‐to‐treat strategy for *H. pylori* infection in the general population may be a promising approach to reduce the burden of gastric cancer. However, *H. pylori* eradication cannot completely protect against gastric cancer, and subjects with increased risk need long‐term surveillance with endoscopy or another modality. In Taiwan, the nationwide availability of endoscopy coverage by insurance means there is a reliable surveillance tool to assess the control of precancerous changes after *H. pylori* eradication and to detect early gastric cancer. These measures will improve survival rates from gastric cancer. The current consensus recommends screening‐to‐test for *H. pylori* infection and endoscopic surveillance for high‐risk groups after *H. pylori* eradication as a cost‐effective method to improve control of gastric cancer.

Statement III‐1: *H. pylori* eradication reduces the incidence of gastric cancer and progression of pre‐malignant gastric lesions. (Evidence level: 1a, Agreement: 100%, Recommendation: A)

More than 80% of gastric cancers are likely due to *H. pylori* infection.^101^ Meta‐analyses and RCTs support *H. pylor*i eradication as treatment for asymptomatic individuals, because such subjects have a 34% lower risk of gastric cancer.^102^, ^103^ Another updated meta‐analysis that examined eight RCTs and 16 cohort studies indicated that *H. pylori* eradication led to a 47% reduced risk of gastric cancer.^104^ This benefit is greater for patients after endoscopic resection of early gastric cancer (54% risk reduction, 95% CI: 40%‐65%) than for asymptomatic individuals (38% risk reduction, 95% CI: 21%‐51%).^104^ Based on the Taiwan National Health Insurance Research Database (NHIRD), early *H. pylori* eradication may decrease the risk of gastric cancer in patients with peptic ulcer disease.^105^ *H. pylori* eradication can reduce and/or reverse the incidence of premalignant gastric lesions.^106^, ^107^, ^108^, ^109^, ^110^ Because appropriate treatment can eradicate *H. pylori* infection in more than 90% of individualsin the general population, we recommend *H. pylori* eradication for prevention of gastric cancer.^109^, ^110^

Statement III‐2: *Helicobacter pylori* eradication reduces the risk of metachronous gastric cancer after tumor resection. (Evidence level: 1b, Agreement: 96%, Recommendation: A)

Because of the significant role of *H. pylori* on gastric carcinogenesis, *H. pylori* should be eradicated in patients with gastric cancer after tumor resection to prevent recurrence. In Japan, the risk for metachronous gastric cancer is lower following *H. pylori* eradication after endoscopic resection of gastric cancer (hazard ratio [HR]: 0.34, 95% CI: 0.16‐0.73).^111^ In addition, a meta‐analysis confirmed a 58% lower risk of metachronous gastric cancer following *H. pylori* eradication after endoscopic resection of gastric cancer.^112^

Statement III‐3: After *H. pylori* eradication, NSAIDs may provide chemoprevention by halting the progression of pre‐malignant lesions, and thereby reduce the risk of gastric cancer; however, their adverse effects should be considered. (Evidence level: 2b, Agreement: 92%, Recommendation: B)

*Helicobacter pylori* infection induces chronic inflammation and a multistage premalignant process during gastric carcinogenesis.^113^ There is upregulation of cyclooxygenase‐2 (COX‐2) in *H. pylori*‐related premalignant lesions, and this persists even after *H. pylori* eradication.^114^ Wong et al. observed that some gastric premalignant lesions regressed after a 24‐month treatment with a selective COX‐2 inhibitor (celecoxib, 200 mg, twice daily)^106^; however, *H pylori* eradication followed by celecoxib treatment had no significant effect on the regression of advanced gastric lesions. Sheu et al. showed that a 12‐month regimen of celecoxib (200 mg daily) improved the regression or prevented the progression of intestinal metaplasia after *H. pylori* eradication.^115^ A meta‐analysis showed that users of aspirin and/or NSAID have reduced risk of gastric cancer.^116^ Based on data from the NHIRD of Taiwan, each year of regular use of an NSAID led to a 21% reduced risk of gastric cancer, and this benefit was greater for patients with *H. pylori* infection.^117^ Based on these observations, chemoprevention using an NSAID after *H. pylori* eradication may be helpful in regression of the premalignant gastric lesions; however, in clinical practice, this approach may lead to more adverse events (such as bleeding) and additional costs. Thus, the benefits and harms must be carefully weighted before translation of these findings into clinical practice.

Statement III‐4: A screen‐to‐treat approach for management of *H. pylori* infection should cost‐effectively reduce the incidence of gastric cancer in intermediate‐ and high‐risk populations. (Evidence level: 2a, Agreement: 88%, Recommendation: B)

The advantage provided by mass screening and eradication of *H. pylori* requires a cost‐benefit analysis. A systemic review of 23 cost‐effectiveness analyses concluded that such a strategy can be cost‐effective.^118^ The initial cost‐effectiveness analyses in Western countries recommended screening to be started in individuals older than 50 years old, because of an incrementally lower cost‐effectiveness ratio in younger individuals.^118^, ^119^, ^120^ In contrast, recent cost‐effectiveness analyses that focused on eastern Asia populations suggested that *H. pylori* eradication was also cost‐effective in younger individuals.^121^, ^122^ A simulation study based on the Taiwan NHIRD suggested the expected years of life lost from gastric cancer would be greater in 30‐year‐olds than 50‐year‐olds.^123^ Thus, the screen‐to‐treat strategy might decrease the incremental cost‐effectiveness ratio if the strategy starts at age 30 in intermediate‐ and high‐risk areas which have high prevalence of *H. pylori* infection.^123^

Statement III‐5: After *H. pylori* eradication, subjects with gastric pre‐malignant lesions still have increased risk of gastric cancer, and thus need endoscopic surveillance at scheduled intervals stratified by the OLGA/OLGIM system. (Evidence level: 2b, Agreement: 96%, Recommendation: A)

Although *H. pylori* eradication can reduce the incidence of gastric cancer, certain subjects remain at risk of gastric cancer after eradication,^124^, ^125^, ^126^ such as those with extensive atrophic gastritis or intestinal metaplasia.^125^, ^126^, ^127^, ^128^, ^129^, ^130^, ^131^, ^132^, ^133^ Reliable staging of premalignant lesions requires a well‐defined biopsy sampling protocol, such as using the updated Sydney system.^134^ Based on the extent and topography of the atrophic gastritis or intestinal metaplasia, two superordinate staging systems, the Operative Link for Gastritis Assessment (OLGA) or Operative Link on Gastric Intestinal Metaplasia Assessment (OLGIM), can assess the risk of gastric cancer in subjects with premalignant lesions.^135^, ^136^ The first‐degree relatives of gastric cancer patients have increased prevalence of advanced‐stage OLGA and OLGIM (III and IV), *H. pylori* infection, and corpus‐predominant gastritis.^135^, ^137^, ^138^, ^139^ A 12‐year prospective cohort study showed that all new gastric neoplasia arose from tissue assessed as high‐stage OLGA.^107^ The annual incidences of gastric cancer are about 0.08% to 0.30% for patients with atrophic gastritis, 0.13% to 0.54% for those with intestinal metaplasia, and 0.39% to 0.56% for those with low‐grade dysplasia.^125^, ^128^, ^129^, ^130^, ^131^, ^132^, ^133^ The 5‐, 10‐, and 15‐year cumulative incidences of gastric cancer among patients with intestinal metaplasia and concurrent dysplasia are 5.6%, 8.7%, and 18.9%, respectively.^133^ Accordingly, surveillance endoscopy should be used to detect early gastric cancer in subjects with premalignant lesions defined by these systems after *H. pylori* eradication.^140^

A surveillance strategy with an endoscopic interval of 1‐3 years is cost‐effective for patients with extensive atrophic gastritis or intestinal metaplasia.^141^, ^142^, ^143^ Thus, a premalignant lesion can be assessed by OLGA/OLGIM stage, and then, the interval for endoscopic surveillance after *H. pylori* eradication can be established to improve control of gastric cancer.

Statement III‐6: Patients with high‐grade gastric intraepithelial neoplasia (dysplasia) should receive endoscopic or surgical resection. (Evidence level: 2b, Agreement: 100%, Recommendation: A)

According to the World Health Organization (WHO), premalignant gastric lesions include adenomas, low‐grade intraepithelial neoplasia (dysplasia), and high‐grade intraepithelial neoplasia (dysplasia).^129^ For patients with high‐grade intraepithelial neoplasia, the progression ranged from 1.4% to 3.3% per year, and nearly 25% of patients developed gastric cancer within 1 year.^130^ In Taiwan, a cohort study reported a HR of 18.8 (95% CI: 9.0‐39.5) for development of gastric cancer from high‐grade intraepithelial neoplasia.^133^ The high rate of progression in patients with high‐grade intraepithelial neoplasia indicates that the neoplasia often contains cancerous foci that are not detected by biopsy. Therefore, we recommend an aggressive strategy, such as endoscopic or surgical resection.

Statement III‐7: Serum pepsinogens, anti‐*H. pylori* antibodies, and certain demographic characteristics are useful in identifying subjects with high risk for gastric cancer. (Evidence level: 2a, Agreement: 92%, Recommendation: B)

Although histological assessment can predict the risk of gastric cancer, this is an invasive approach. Serological tests can serve as alternative methods for mass screening and predicting the risk of gastric cancer.^144^ A meta‐analysis estimated that the sensitivity was 77.3% and the specificity was 73.2% when using pepsinogen I (≤70 ng/mL) and the pepsinogen I/II ratio (≤3) to predict gastric cancer.^145^ Especially in cohorts from eastern Asia, the pepsinogen test and *H. pylori* antibodies were significant predictors for gastric cancer.^146^ Based on analysis of the Taiwan NHIRD, researchers have developed a risk assessment method with demographic factors (age, sex, peptic ulcer site, peptic ulcer complications, *H. pylori* eradication, and NSAID usage) to predict the 1‐year and 2‐year risks of gastric cancer.^147^ Application of a nomogram allowed division of subjects into quartiles, based on predicted risk scores, and the cumulative incidences of gastric cancer at 1 year increased from the lowest to the highest quartile (7.4, 14.2, 25.5, and 86.6 per 10 000 people).

## 4 Dissemination Strategies and Legal Issues

These statements are based on the best available evidence to pursue better quality of care and will be updated every 5 years. They are not suitable for deciding the standard of care for specific cases. This consensus statement will be disseminated by (1) presentation at the 2017 annual meeting of our society during Taiwan Digestive Week (Taipei, Taiwan); (2) electronic and paper format to national societies/associations of gastroenterologists for their iterations; and (3) on the website of our society.

## Author Contributions

Dr. Sheu BS coordinated as the chairman of the Taiwan expert group to compose the draft of the manuscript. Dr. Wu MS served as the co‐chairman, Dr. Chiu CT, Lo GH, Wu DC, Liou CM, Wu CY, Cheng HC, Lee YC, Hsu PI, Chang CC, and Chang WL reviewed the literatures and statements. Dr. Lin JT applied the funding for the expert meeting and critically reviewed the article.
