# Supplementary material for: Consensus on the clinical management, screening‐to‐treat, and surveillance of Helicobacter pylori infection to improve gastric cancer control on a nationwide scale
Source: Helicobacter. 2017 Jan 8;22(3):e12368. doi: 10.1111/hel.12368 (PMC5434958; doi:10.1111/hel.12368)
Supplement: Supplementary file 1 [file HEL-22-na-s001.docx]

**The Expert Group of the Taiwan Helicobacter Consensus Meeting**

Jaw-Town Lin (*Fu Jen Catholic University, New Taipei City, Taiwan),* Bor-Shyang Sheu (*National Cheng Kung University Hospital and College of Medicine, National Cheng Kung University, Tainan, Taiwan),* Ming-Shiang Wu (*National Taiwan University Hospital, Taipei, Taiwan),* Cheng-Tang Chiu (*Chang Gung Memorial Hospital, Linko, Taiwan),* Jiing-Chyuan Luo (*Taipei Veterans General Hospital, Taipei, Taiwan),* Chun-Che Lin (Chung Shan Medical University, Taichung, Taiwan), Chi-Sen Chang (*Taichung Veterans General Hospital, Taichung, Taiwan),* Ming-Jen Sheu (Chi-Mei Medical Center, Tainan, Taiwan), Deng-Chyang Wu (*Kaohsiung Medical University Hospital, Kaohsiung, Taiwan),* Jyh-Ming Liou (*National Taiwan University Hospital, Taipei, Taiwan),* Chao-Hung Kuo (*Kaohsiung Medical University Hospital, Kaohsiung, Taiwan),* Seng-Kee Chuah (Kaohsiung Chang Gung Memorial Hospital, Kaohsiung, Taiwan), Chun-Ying Wu (*Taichung Veterans General Hospital, Taichung, Taiwan),* Chia-Long Lee (Cathay General Hospital Medical Center, Taipei, Taiwan), Yi-Chia Lee (*National Taiwan University Hospital, Taipei, Taiwan),* Hsiu-Chi Cheng (*National Cheng Kung University Hospital and College of Medicine, National Cheng Kung University, Tainan, Taiwan),* Ming-Jong Bair (Mackay Memorial Hospital, Taitung Branch, Taitung, Taiwan), Chih-Hsun Yi (Hualien Tzu Chi Hospital, Buddhist Tzu Chi Medical Foundation and Tzu Chi University , Hualien , Taiwan), Chi-Yang Chang ( E-Da Hospital, I-Shou University, Kaohsiung, Taiwan), Ping-I Hsu (*Kaohsiung, Veterans General Hospital, Kaohsiung, Taiwan),* Chun-Chao Chang (Taipei Medical University Hospital and School of Medicine, College of Medicine, Taipei Medical University, Taipei, Taiwan), Wei-Lun Chang (*National Cheng Kung University Hospital and College of Medicine, National Cheng Kung University, Tainan, Taiwan),*

Rong-Yaun Shyu (Buddhist Tzu Chi General Hospital Taipei Branch, New Taipei City, Taiwan), Yao-Jong Yang (*National Cheng Kung University Hospital and College of Medicine, National Cheng Kung University, Tainan, Taiwan), and* Kuan-Yang Chen (Ren-Ai Branch, Taipei City Hospital, Taipei, Taiwan).
